# Are You Your Friends’ Friend? Poor Perception of Friendship Ties Limits the Ability to Promote Behavioral Change

**DOI:** 10.1371/journal.pone.0151588

**Published:** 2016-03-22

**Authors:** Abdullah Almaatouq, Laura Radaelli, Alex Pentland, Erez Shmueli

**Affiliations:** 1 Center for Computational Engineering, Massachusetts Institute of Technology, Cambridge, Massachusetts, United States of America; 2 Media Lab, Massachusetts Institute of Technology, Cambridge, Massachusetts, United States of America; 3 Department of Industrial Engineering, Tel-Aviv University, Ramat Aviv, Tel-Aviv, Israel; 4 Institute for Data, Systems, and Society (IDSS), Massachusetts Institute of Technology, Cambridge, Massachusetts, United States of America; Hangzhou Normal University, CHINA

## Abstract

Persuasion is at the core of norm creation, emergence of collective action, and solutions to ‘tragedy of the commons’ problems. In this paper, we show that the directionality of friendship ties affect the extent to which individuals can influence the behavior of each other. Moreover, we find that people are typically poor at perceiving the directionality of their friendship ties and that this can significantly limit their ability to engage in cooperative arrangements. This could lead to failures in establishing compatible norms, acting together, finding compromise solutions, and persuading others to act. We then suggest strategies to overcome this limitation by using two topological characteristics of the perceived friendship network. The findings of this paper have significant consequences for designing interventions that seek to harness social influence for collective action.

## Introduction

Studies of collective action have shown that social influence can greatly facilitate mobilization around collective goods, predict success of the action, and guide its design [1]. For instance, social influence is found to be a key factor in political uprisings [2, 3], strike movements [3], and other types of participation [4]. Social influence in networks is also a well-recognized key factor in the diffusion of new behaviors [5–8], new ideas [9] and new products [10, 11] in society.

Moreover, in recent years, peer-support programs are emerging as highly effective and empowering ways to leverage peer influence to support behavioral change of people [12]. One specific type of peer-support programs is the “buddy system”, in which individuals are paired with another person (i.e., a buddy) with the responsibility to support their attempt to change their behavior. Such a system has been used to shape people’s behavior in various domains including smoking cessation [13], weight loss [14], diabetes management [15] or alcohol misuse.

Consequently, the need to understand the factors that impact the level of influence individuals exert on one another is of great practical importance. Recent studies have investigated how the effectiveness of peer influence is affected by different social and structural network properties, such as clustering of ties [5], similarity between social contacts [6], and the strength of ties [16]. However, how the effectiveness of social influence is affected by the reciprocity and directionality of friendship ties is still poorly understood.

Individuals commonly assume their affective relationships to be reciprocal by default [17, 18]. For instance, when one considers another individual as “friend”, the common expectation is that this other individual also thinks of them as friends. Moreover, reciprocity is implicitly assumed in many scientific studies of friendship networks by, for example, marking two individuals as being friends or not being friends (e.g., [19–22]). Despite this common expectation, in reality not all friendships are reciprocal [23]. When analyzing self-reported relationship surveys from several experiments, we find that the vast majority of friendships are expected to be reciprocal, while in reality, only about half of them are indeed reciprocal. These findings suggest a profound inability of people to perceive friendship reciprocity, perhaps because the possibility of non-reciprocal friendship challenges one’s self-image.

We further show that the asymmetry in friendship relationships has a large effect on the ability of an individual to persuade others to change their behavior. Moreover, we show that the effect of directionality is larger than the effect of the self-reported strength of a friendship tie [16] and thus of the implied ‘social capital’ of a relationship. Our experimental evidence comes through analysis of a fitness and physical activity intervention, in which subjects were exposed to different peer pressure mechanisms, and physical activity information was collected passively by smartphones. In this experiment, we find that effective behavioral change occurs when subjects share reciprocal ties, or when a unilateral friendship tie exists from the person applying the peer pressure to the subject receiving the pressure, but not when the friendship tie is from the subject to the person applying peer pressure.

Our findings suggest that this misperception of friendships’ character for the majority of people may result in misallocation of efforts when trying to promote a behavioral change. For instance, in the smoking cessation example mentioned above, our results suggest that the reciprocity and directionality of the friendship relationship between the smoker and the buddy can greatly affect the success of the intervention, and therefore the intervention designers cannot rely on how the smoker perceive his/her relationships with the buddies.

To overcome this limitation, we show that two topological features of the perceived friendship network—social embeddedness and social centrality—can alone effectively identify the most likely targets for effective behavioral change. As a consequence, people seeking to shape the behavior of others could become more effective by relying on these features rather than on the perceived character of the relationship itself. Revising our smoking cessation example, the intervention designers can leverage some topological features of the perceived friendship network of the smokers to select buddies that would induce more effective peer-pressure.

## Results

### Friendship Directionality

We provide a quantitative assessment of people’s expectation on the reciprocity of their friendship relationships through a self-reported survey that we collected among 84 students of an undergraduate course (see Reciprocity Survey in S1 File). Similarly to other self-reported friendship surveys (see for example [24–26]), we asked each participant to score every other participant on a 0–5 scale, where 0 means “I do not know this person”, 3 means “Friend” and 5 means “One of my best friends.” In addition, participants were also asked to ‘predict’ how other participants would score them. Participants in the experiment were early career (age 23–38) adults taking a university course in applied management. Gender balance was 40% male and 60% female. This age range and gender balance is similar to participants in the FunFit experiment [24]. The study was approved by the Institutional Review Board (IRB) and conducted under strict protocol guidelines (see Reciprocity Survey in S1 File).

**Fig 1:**
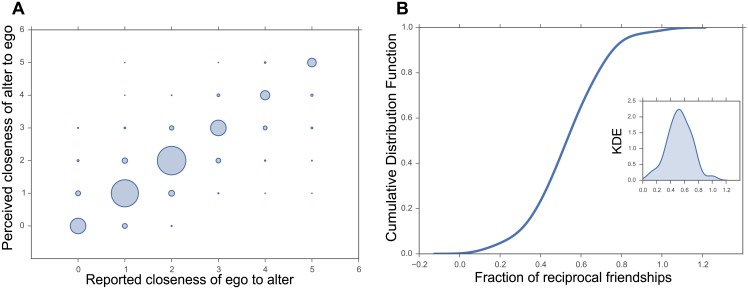
Reciprocity expectations. (A) The perceived closeness score of nominated alter to nominating ego vs. the reported closeness score of nominating ego to nominated alter. The size of each circle represents the number of edges with the specific perceived and reported closeness scores. (B) Distribution (Estimated Cumulative Distribution Function and Kernel Density Estimate) of the fraction of reciprocal friendships at the single participant level.

Examining the relationship between how each subject scored the other subjects and his/her perception of how the other subjects would have scored him/her reveals a very strong and significant correlation (*r* = 0.95, *p* = 0). Fig 1 shows this high expectation for reciprocity in friendship scores. In fact, in 94% (1273 out of 1353) of the cases in which a subject nominated another subject as a friend (i.e., closeness score >2), he/she also expected the other subject to nominate him/her back as a friend.

In contrast to the high expectations of reciprocity among the participants, we find that almost half of the friendships are actually non-reciprocal. We show this by constructing a directional friendship network based on explicit friendship nominations (i.e., closeness scores >2). In this network, we consider a friendship tie to be “reciprocal” when both participants identify each other as friends. Alternatively, the tie is “unilateral” when only one of the participants identifies the other as a friend. The final directed friendship network consists of 84 nodes (i.e., participants) and 775 edges (i.e., explicit friendships). Examining the relationship between the reported friendship scores on the two sides of these edges reveals a relatively weak correlation (*r* = 0.36, *p* = 0). Furthermore, only half (i.e., 53%) of these edges are indeed reciprocal (413 out of 775).

In order to rule out the possibility that there were a few participants that nominated a large number of other participants as friends (high out-degree) and were not nominated back as friends by many others (low in-degree) and therefore skewed the global fraction of reciprocal friendships, we further calculated the fraction of reciprocal friendships at the single participant level. We find the distribution of these fractions for all participants to be normal and centered around 0.5 (see Fig 1). Therefore the overall result is not skewed by a few outlier individuals.

We find this result to be consistent across many self-reported friendship networks that we have analyzed: only 45% (315 out of 698) of friendships are reciprocal in the Friends and Family dataset [24], 34% (28 out of 82) in the Reality Mining dataset [20], 35% (555 out of 1596) in the Social Evolution dataset [25], 49% (102 out of 208) in the Strongest Ties dataset [26], and 53% (1683 out of 3160) in the Personality Survey. The first three surveys were collected at an American university, the fourth at a European university, and the latter at a Middle Eastern university (see S1 File).

Similarly, a previous study [23] in which adolescents were asked to nominate at most 10 of their best school friends (5 male and 5 female) found that only 64% of the reported friendships were indeed reciprocal. Our findings reinforce this finding by investigating multiple datasets from three continents, and by using complete nomination networks (in which each participant is asked about every other participant), resulting in an even more prominent lack of reciprocity. The phenomenon of frequent unreciprocated friendships may be tied to the prevalence of social status and power hierarchy. This suggests that many of the non-reciprocal friendships are aspirational: people want to be friends with higher-status individuals and behave in ways that indicate friendship (e.g., naming them as friends), but higher-status individuals have greater choice in which friendships to reciprocate and choose to only behave as a friend to a subset of the friendships offered to them. In the study of reciprocal friendships in US high schools, the first-generation and black children were found to have many fewer reciprocal friendships, which may indicate that these social groups have stronger hierarchical social structure [27, 28]. Moreover, to the best of our knowledge, this is the first study to analyze the expectations that individuals have from their friendship relationships and to compare the expected and actual relationships.

### Directionality and Induced Peer-Pressure

Social scientists have long believed that reciprocal friendships are more intimate [23, 29, 30], provide higher emotional support [30–33], and form a superior resource of social capital [23, 34, 35] when compared to those that are not reciprocated. This holds whether or not any party of the dyad is aware of the reciprocity status of their relationship [23].

In order to understand the effect of reciprocal ties on peer-pressure, we turn to the Friends and Family study. In addition to surveys that were used to determine the closeness of relationships among participants (similarly to the Reciprocity Survey above), it included a fitness and physical activity experimental intervention. The study (Approval#: 0911003551) was reviewed and approved by the Committee on the Use of Humans as Experimental Subjects (COUHES) at MIT. All participants provided a written consent to participate in this study and COUHES approved the consent procedure.

**Fig 2:**
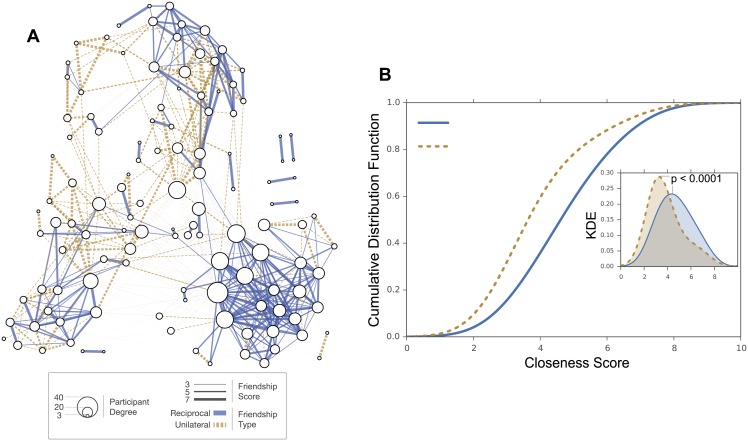
Friends and Family friendship network. (A) The undirected friendship nomination network in the Friends and Family study, where nodes represent participants and edges represent explicit friendship ties (only ties with a closeness score >2 are considered). The size of a node is proportional to its degree. The width of an edge represents the closeness score for unilateral ties and the average closeness score for reciprocal ties (average scores are used for visualization purposes only, for the analysis, reciprocal ties are considered as two separate ties). The line style of an edge represents the type of the edge, where reciprocal ties are solid lines and unilateral ties are dashed lines. (B) The Cumulative Distribution Function (CDF) and Kernel Density Estimate (KDE) of closeness scores are computed for unilateral ties (dashed line) and reciprocal ties (solid line). Note that due to the nature of the Gaussian KDE process, it is possible that the estimation extends past the largest and smallest values in the dataset.

As part of the closeness surveys, each participant scored other participants on a 0–7 scale, where a score of 0 meant that the participant was not familiar with the other, and 7 that the participant was very close to the other. Analyzing the distribution of closeness scores associated with the two types of ties in the Friends and Family friendship network (see Fig 2) reveals that participants that share a reciprocal friendship tend to score each other higher (average of 4.7) in terms of closeness when compared to participants that share unilateral friendship (average of 3.9) (*p* < 10^−4^, two-sample t-test).

However, we hypothesize that ‘reciprocity’ and ‘directionality’ of friendships may be critical factors in promoting peer influence, beyond the mere effect of the total tie ‘strength’ bound up in the relationship.

To support our hypothesis, we investigate the FunFit experiment—a fitness and physical activity experimental intervention—conducted within the Friends and Family study population during October to December of 2010. The experiment was presented to participants as a wellness game to help them increase their daily activity levels. Subjects received an ‘activity app’ for their mobile phone which passively collected their physical activity data and showed the participants how their activity level had changed relative to their previous activity level, and the amount of money they had earned by being more active. 108 out of the 123 active Friends and Family subjects at that time elected to participate and were allocated into three experimental conditions, allowing us to isolate different incentive mechanisms varying monetary reward, the value of social information, and social pressure/influence:

- **Control**: subjects were shown their own progress and were given a monetary reward based on their own progress in increasing physical activity relative to the previous week.
- **Peer See**: subjects were shown their own progress and the progress of two “buddies” in the same experimental group, and were given a monetary reward based on their own progress in increasing physical activity relative to the previous week.
- **Peer Reward**: subjects were shown their own progress and the progress of two “buddies” in the same experimental group, but their rewards depended only on the progress of the two “buddies”. This condition realizes a social mechanism based on inducing peer-to-peer interactions and peer pressure [36].

However, for the purpose of our analysis in this section, we combine the samples from the two peer pressure treatments and omit the control group.

During the initial 23 days of the experiment (Oct 5–Oct 27), denoted as P1, the baseline activity levels of the subjects were collected. The actual intervention period is denoted as P2. During the intervention period, the subjects were given feedback on their performance in the form of a monetary reward. The monetary reward was calculated as a function of the subject’s activity data relative to the previous week and was divided according to the subject’s experimental condition (i.e., Peer See and Peer Reward). Noting that the physical activity was measured passively by logging the smartphone accelerometer (as opposed to self-reported surveys) and the game was not designed as a competition, every subject had the potential to earn the maximal reward. That is, a previously non-active participant could gain the same reward as a highly active one, while the highly active person would need to work harder.

The results in [24] show that the two social conditions (i.e. Peer See and Peer Reward) do significantly better than the control group. Furthermore, the results suggest that there is a complex contagion effect [37], due to the reinforcement of the behavior from multiple social contacts [5, 37], related to pre-existing social ties between participants. Our analysis here focuses on the role of reciprocity and directionality of friendship ties in this contagion process.

In order to investigate the role of reciprocity and directionality of friendship ties in the contagion process, we performed a regression analysis in which the dependent variable was the change in physical activity between the post-intervention phase and the pre-intervention phase (i.e., the average daily physical activity in P2 divided by the average daily physical activity in P1). Model specifications are detailed in S1 File.

For our study, we refer to a participant whose behavior is being analyzed as “ego”, and participants connected to the ego (i.e., experimental “buddies”) are referred to as “alters”. Because friendship nominations are directional, we studied the three possible types of friendships (from the prospective of the ego) as independent variables: an “ego perceived friend”, in which an alter identifies an ego as a friend (i.e., incoming tie); an “alter perceived friend” in which an ego identifies an alter as a friend (i.e., outgoing tie); and a “reciprocal friend”, in which the identification is bidirectional (i.e., reciprocal tie). Finally, we also included the tie strength (i.e., the sum of the closeness scores between an ego and his or her alters) as a control variable, which has been previously investigated as a moderator of the effect of social influence [16].

Table 1 reports the effects found in our regression analysis (recall that the dependent variable in our model is the change in activity for the egos). We find that the reciprocity and directionality of a friendship have an effect on the amount of induced peer pressure, and these effects are much larger than the total tie strength.

**Table 1:** Friendship types effect the strength of peer pressure. Change in physical activity under experiment conditions shows that the type of friendship (e.g., reciprocity and directionality) have an effect on the amount of induced peer pressure and the effectiveness of the intervention.

	Model 1	Model 2	Model 3
Constant (Intercept)	0.05 (0.08)	0.63*** (0.11)	0.55* (0.26)
Reciprocal friend	0.44** (0.16)	0.30* (0.13)	0.33* (0.14)
Alter perceived friend	0.15 (0.13)	0.12 (0.11)	0.13 (0.12)
Ego perceived friend	0.31* (0.12)	0.24* (0.10)	0.24* (0.10)
Tie Strength	−0.04^⋅^ (0.02)	−0.03^⋅^ (0.02)	−0.03^⋅^ (0.02)
Pre-intervention activity		−0.43*** (0.07)	−0.43*** (0.07)
Ego gender			0.08 (0.07)
Same-gender friend			0.01 (0.05)
Ego age			0.00 (0.01)
Same-ethnicity friend			0.03 (0.05)
R^2^	0.16	0.46	0.47
Adj. R^2^	0.11	0.42	0.40
Num. obs.	76	76	76

Dependent variables significance levels:

*** *p* < 0.001,

** *p* < 0.01,

* *p* < 0.05,

^⋅^ *p* < 0.1.

Standard errors in brackets.

The strongest effect for both treatment groups (*N* = 76) in this study was found for the reciprocal factor (*p* < 0.01; Model 1) even when controlling over the strength of the tie (the tie strength is weakly significant *p* = 0.07). That is, alters in reciprocal friendships have more of an effect on the ego than alters in other types of friendships.

Interestingly, when the ego was perceived as a friend by the alters (i.e., incoming edges from the alters to the ego), the effect was also found to be positive and significant (*p* < 0.05). On the other hand, no statistically significant effect was found when the alters were perceived as friends by the ego (i.e., outgoing edges from the ego to the alters). Therefore, the amount of influence exerted by individuals on their peers in unilateral friendship ties seems to be dependent on the direction of the friendship.

Unlike previous works on social contagion effects [7, 38], which were conducted without peer-to-peer incentives, we find that influence does not flow from nominated alter to nominating ego. Surprisingly, alter’s perception of ego as a friend would increase alter’s ability to influence ego’s behavior when ego does not reciprocate the friendship. We attribute this difference to the fact that there is a peer-to-peer incentive mechanism, and therefore there are likely to be differences in communication when the alters believe the ego to be their friend versus when they do not.

We find these results to be consistent even when including additional detailed controls for the ego’s age, gender, whether the buddies are from the same or opposite gender, whether the buddies have the same ethnicity, and their pre-intervention activity levels (Model 2 & Model 3). We find the pre-intervention activity to be the only control variable with a significant effect. Additional analyses and robustness tests can be found in the S1 File.

### Predicting Directionality

Previous studies have investigated numerous factors that could have an influence on the reciprocity of friendships. This would include socio-economic status [39], gender differences [40] and ethnic or racial origin [41].

Here we are interested in the predictability of reciprocity (i.e., reciprocal versus unilateral) and directionality (i.e., incoming versus outgoing edge) of ties based on the topological structure of the underlying *undirected* and *unweighted* social network, independently of additional information such as gender, race, tie strength, etc. Such additional information is often not available when analyzing communication networks [20, 42], trust networks [43], or similar data. Therefore, it is important to know the structural characteristics that allow effective intervention strategies.

Two possible social factors that can be used to predict the reciprocity and directionality of a friendship tie between two individuals are: (i) Social Embeddedness: the extent to which their friendship circles overlap; and (ii) Social Centrality: the difference in their social hierarchical organizational status.

**Fig 3:**
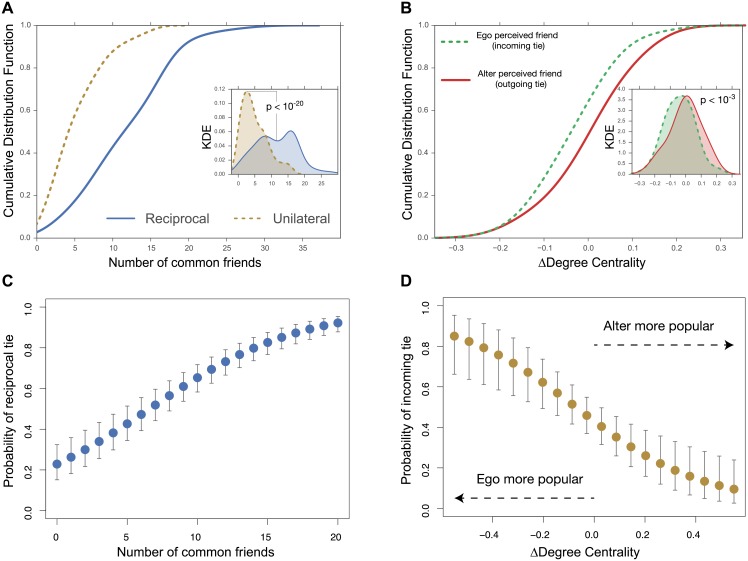
The effect of Social Embeddendness (SE) and the difference in Social Centrality (SC) on determining the tie type in the Friends and Family dataset. In Panels (A) and (B), we observe that SE (i.e., represented by the number of common friends) and SC (as Δ Degree centrality) are good discriminators between reciprocal and unilateral ties, as well as between the two directions of unilateral ties. Panels (C) and (D) show the effect of SE and SC on the Probability of an ego to form a reciprocal tie or be perceived as a friend, respectively. Control variables include the ethnic and racial origin as well as religious and gender differences. The vertical gray bars for both panels show 95% confidence intervals based on 10,000 drawn sets of estimates from the coefficient covariance matrix and with all other covariates held at their median. Additional information is available in the S1 File.

The Social Embeddedness (SE) of a pair of individuals captures the idea that the behavior individuals choose are significantly constrained by the social relations within which they function [44]. Inspired by this idea, we expect social embeddedness to explain reciprocity as an effect of the network transitivity property [45]. Here, we use the number of common friends the two individuals share to capture their SE in the network. The finding that friends share more common neighbors has been extensively studied in the literature (e.g., [42, 46–48]). Fig 3 highlights the effect of SE on the probability of an ego to form a reciprocal tie in the Friends and Family dataset while holding demographic covariates (e.g., gender differences, and ethnic and racial origin) at their median values (see S1 File). This result supports our hypothesis by showing that reciprocal ties exhibit higher average number of common friends. We have also investigated five additional measures of SE and show that they perform similarly (see S1 File).

We also look at the difference in Social Centrality (SC) as a possible explanation of the directionality of unilateral friendship ties. The Social Centrality (SC) of an individual captures the idea that hierarchical social organizations are highly characterized by ordered, linearly transitive social relationships [49, 50]. Therefore, one would expect that the direction of unilateral ties would tend to flow from individuals with lower status to individuals with higher status [50]. Here, we use the *Degree Centrality* measure to capture the centrality of a node in the network (we investigate three other common measures of SC in the S1 File and show that they generate similar results). Fig 3 supports our hypothesis by showing that unilateral ties tend to flow from the less central node to the more central node of the dyad. We find the effect to be persistent after controlling for the demographic attributes.

The conditional entropy for the two measures, H(reciprocity|number of common friends) = 1.8 and H(directionality|Δdegree centrality) = 2.02568, which implies good separation of the classes and that good classification is possible. To confirm this, we further analyzed the predictive power of these measures in the two classification tasks: (i) reciprocal vs unilateral ties; and (ii) the direction of unilateral ties. For each classification task, we train and test a classifier in K-fold-cross validation (*K* = 10). First, we use a simple Logistic Regression classifier and a single feature for each classification task (i.e. number of common friends for the first task and difference in degree centrality for the second task). When trying to identify reciprocal ties, the Logistic Regression classifier performed significantly better than the baseline, obtaining 0.81 AUC (95% CI: 0.77–0.85). As for identifying the direction of unilateral ties, the Logistic Regression classifier performed better than the baseline, although the results obtained were less compelling: an average AUC of 0.61 (95% CI: 0.58–0.66). These results are consistent with the results of the conditional entropy, where the first task (i.e., predicting reciprocity) is more attainable than the second (i.e., predicting directionality).

**Fig 4:**
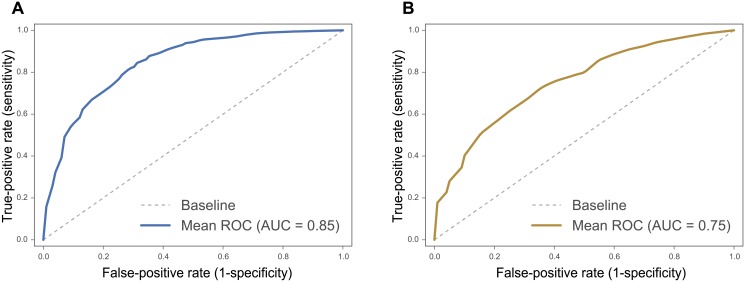
The classification performance. Mean ROC curves demonstrating the model performance in predicting ties type. Panel (A) shows the prediction performance for reciprocal ties (AUC = 0.85, 95% CI: 0.82–0.87). Panel (B) shows the model performance in prediction incoming ties (AUC = 0.75, 95% CI: 0.70–0.80).

Finally, we used all 10 identified features (6 SE features and 4 SC features as described in the S1 File to train a Random Forest classifier for each classification task, obtaining encouraging results for both classification tasks: an average AUC of 0.85 (95% CI: 0.82–0.87) for the reciprocal classification task and an average AUC of 0.75 (95% CI: 0.70–0.80) for the directionality task. The performances of the two classification tasks are reported in Fig 4. We further experimented with five additional friendship nomination networks, and both prediction tasks showed similar results (see details in the S1 File).

## Discussion

In this paper we have demonstrated the important role that reciprocity and directionality of friendship ties play in inducing effective social persuasion. We have also shown that individuals have difficulty in judging the reciprocity and directionality of their friendship ties (i.e., how others perceive them), and that this can be a major limiting factor for the success of cooperative arrangements such as peer-support programs.

For instance, in [8, 51] it was shown that the assumption that individuals with a high number of incoming friendship nominations are ‘influencers’ is flawed, and that such people are no better and often worse than average people at exerting social influence. Our results suggest that this is because many of those ties are either not reciprocal or are in the wrong direction to be effective at persuasion.

Finally, we investigated the predictability of friendship ties in friendship networks with respect to two categories of features, namely, *Social Embeddedness* and *Social Centrality*, and showed that these features can be used to markedly improve judgments about the reciprocity and directionality of friendship ties.

Previous studies have found that people tend to adopt the behaviors of peers that they are passively exposed to, with the explicit self-reported friends and intimate social acquaintances playing a peripheral role (e.g., [5, 52]). Other studies have shown that passive exposures to peer behavior can increase the chances of becoming obese [7, 52], registering for a health forum Web site [5], signing up for an Internet-based diet diary [6], or adopting computer applications [10]. However, our results suggest a fundamental difference between how social learning (i.e., passive exposure) and social persuasion (i.e., active engagement) spread behaviors from one person to another. Indeed we see this as a trend in our data, with participants in the active Peer Reward condition showing more of the directionality effect than those in the Peer See condition (see S1 File for additional details). This is consistent with the effect of the incentive mechanism: the Peer See condition induces social pressure only through social comparison, whereas in the Peer Reward condition there is a monetary reward in addition to the pressure induced by social comparison.

Although we demonstrated the ability to predict tie direction and reciprocity from Social Embeddedness and Difference in Social Status, these measures are also likely to constrain influence, independently of directionality, via mechanisms such as social reinforcement, tie strength, and social status. Therefore, these potential confounding effects need to be taken into account when moving from an experimental to field setting.

The findings of this paper have significant consequences for designing interventions that seek to harness social influence for collective action. This paper also has significant implications for research into peer pressure, social influence, and information diffusion as these studies have typically assumed undirected (reciprocal) friendship networks, and may have missed the role that the directionality of friendship ties plays in social influence. This suggests that both practitioners and researchers should consider the reciprocity and directionality of friendship relationships when thinking about behavior change due to social influence.

## Supporting Information

S1 File Contains the following. Sections: 1 The Friends and Family Study, 2 The FunFit Experiment, 3 The Reciprocity Survey, 4 Directionality and Induced Peer Pressure, 5 Social Embeddedness, 6 Social Centrality, 7 Predicting Reciprocity and Directionality, 8 Reciprocal Ties and Incoming Edges Factors, 9 Additional Datasets. Table S1: numbers of participants per period/condition. Table S2: demographics of participants in the reciprocity survey. Table S3: regression coefficients for the change in activity under different experiment conditions and controls. Table S4: regression coefficients for the change in physical activity for peer-see and peer-reward intervention groups. Table S5: logit coefficients for the reciprocal ties and incoming unilateral ties with controls. Table S6: the evaluation results of reciprocity and directionality classification on additional datasets. Figure S1: Probability density function of the tie strength in the Peer-View and Peer-Reward groups. Figure S2: Change in physical activity under experiment conditions. Figure S3: Social Embeddedness features. Figure S4: Social Centrality features. Figure S5: Mean ROC curves demonstrating the model performance in predicting ties type. Figure S6: Mean ROC for Social Embeddedness features. Figure S7: Mean ROC for Social Centrality features. Figure S8: Fraction of reciprocal ties in additional datasets. Figure S9: Comparison of classification results with additional datasets.
(PDF)

S1 Datasets The reciprocity survey data. (CSV)
