# Supplementary material for: Are You Your Friends’ Friend? Poor Perception of Friendship Ties Limits the Ability to Promote Behavioral Change
Source: PLoS One. 2016 Mar 22;11(3):e0151588. doi: 10.1371/journal.pone.0151588 (PMC4803335; doi:10.1371/journal.pone.0151588)
Supplement: S1 File — Sections: 1 The Friends and Family Study, 2 The FunFit Experiment, 3 The Reciprocity Survey, 4 Directionality and Induced Peer Pressure, 5 Social Embeddedness, 6 Social Centrality, 7 Predicting Reciprocity and Directionality, 8 Reciprocal Ties and Incoming Edges Factors, 9 Additional Datasets. Table S1: numbers of participants per period/condition. Table S2: demographics of participants in the reciprocity survey. Table S3: regression coefficients for the change in activity under different experiment conditions and controls. Table S4: regression coefficients for the change in physical activity for peer-see and peer-reward intervention groups. Table S5: logit coefficients for the reciprocal ties and incoming unilateral ties with controls. Table S6: the evaluation results of reciprocity and directionality classification on additional datasets. Figure S1: Probability density function of the tie strength in the Peer-View and Peer-Reward groups. Figure S2: Change in physical activity under experiment conditions. Figure S3: Social Embeddedness features. Figure S4: Social Centrality features. Figure S5: Mean ROC curves demonstrating the model performance in predicting ties type. Figure S6: Mean ROC for Social Embeddedness features. Figure S7: Mean ROC for Social Centrality features. Figure S8: Fraction of reciprocal ties in additional datasets. Figure S9: Comparison of classification results with additional datasets. (PDF) [file pone.0151588.s001.pdf]

Supplementary Materials for: Are You Your Friends' Friend? Poor Perception of Friendship Ties Limits The Ability to Promote Behavioral Change

Abdullah Almaatouq,[1,2*] Laura Radaelli,[2,3] Alex (Sandy) Pentland,[2,4] Erez Shmueli[2,3]

[1]Center for Computational Engineering, Massachusetts Institute of Technology

[2]The Media Lab, Massachusetts Institute of Technology,

[3]Department of Industrial Engineering, Tel-Aviv University

[4]Institute for Data, Systems, and Society (IDSS), Massachusetts Institute of Technology

[*]To whom correspondence should be addressed; E-mail: amaatouq@mit.edu

1 The Friends and Family Study

The Friends and Family dataset [1] is based on a year long study, which collected an immensely rich and dense information on the lives of its 130 participants (approximately 64 families).

Subject Pool The participants were all members of a young-family residential living community at a major North American university, where at least one of the members of each family was affiliated with the university. The entire residential community was composed of over 400 residents (approximately half of which had children), and exhibited a lot of friendship ties between its members. Compared with previous social computing observatory studies [2, 3], the Friends and Family community includes a more diverse subject pool and provides a unique perspective into a phase of life that has not been traditionally studied in the field of ubiquitous computing - married couples and young families.

Mobile Phone Logging Software Each participant in the study was equipped with an Android-based mobile phone incorporating the "Funf" platform. This platform is essentially a passive sensing software explicitly designed to continuously collect over 25 phone-based signals, including location, accelerometry, Bluetooth (BT) based device proximity, communication activ-

ities, installed applications, currently running applications, multimedia and file system information, and additional data generated by dedicated experimental applications.

Self-Reported Survey Data In addition to the passive data collection, participants completed surveys at regular intervals. Monthly surveys included questions about self-perception of relationships, group affiliation, interactions, and also standard scales like the Big-Five personality test. Daily surveys included questions like mood, stress, sleep, productivity, socialization and others.

Additionally Collected Data Participants could also opt to provide information on: (i) their purchases through submission of receipts and credit card statements; and (ii) their online socialization activities through the installation of a Facebook app (approximately 70% of subjects opted to install the app).

Incentive Participants committed to use the study phone as their primary phone for the duration of the study and in return they were able to keep the phone at the end of the study. In addition, participants were extra compensated for every out-of-routine task, such as filling out surveys, submitting receipts or participating in interventions.

Human Subjects Approval The study was reviewed and approved by the Committee on the Use of Humans as Experimental Subjects (COUHES) with the approval number 0911003551. COUHES is responsible for reviewing and approving all research involving human subjects that is performed under the auspices of MIT. All participants provided a written consent to participate in this study and COUHES approved the consent procedure. The study was conducted under strict protocol guidelines. The protection of participants' privacy and sensitive information was a key consideration: data were linked to coded identifiers of the participants and not to their real world personal identifiers; all human-readable text, such as phone numbers and text messages, was captured as hashed identifiers, and was never stored in its clear-text form and collected data were physically secured and de-identified before being aggregated for analysis. A second important consideration was to be as unobtrusive as possible to the subjects' life routines.

2 The FunFit Experiment

FunFit is a fitness and physical activity experimental intervention conducted within the Friend and Family study from October 2010 to December 2010. The main goal of the experiment was to explore the question of understanding social influence and motivation in the context of health and wellness activities. The experiment was presented to participants as a wellness game to help them increase their daily activity levels. 108 out of the 123 active Friends and Family subjects at that time elected to participate and were allocated into three experimental conditions,

allowing us to isolate different incentive mechanisms related to monetary reward, the value of social information, and social pressure/influence:

- **Control:** subjects were shown their own progress and were given reward based on their own progress in physical activity.
- **Peer See:** subjects were shown their own progress and the progress of two "buddies" in the same experimental group, and were given reward based on their own progress in physical activity.
- **Peer Reward:** subjects were shown their own progress and the progress of two "buddies" in the same experimental group, but their rewards depended only on the progress of the two buddies. This condition simulates a social mechanism based on inducing peer-to-peer interactions and peer pressure.

The allocation algorithm was designed to pair each participant in the Peer-View and Peer-Reward with two buddies within their group, while (i) prioritizing pairings with closer friends, (ii) eliminating pairings of spouses, and (iii) eliminating reciprocal pairings of buddies. The algorithm was formulated as an integer programming optimization problem and was solved by applying an iterative heuristic (see [1] for further details). The allocation algorithm resulted in a highly variable distribution of tie strength as shown in Fig. S1.

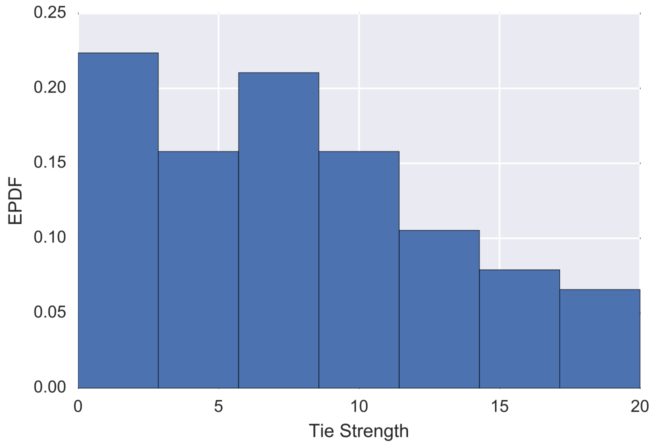

Figure S1: Probability density function of the tie strength in the Peer-View and Peer-Reward groups. Tie strength is computed as the sum of the closeness scores between the subject and the buddies.

During the initial 23 days of the experiment (Oct 5 - Oct 27), denoted as P1, the baseline activity levels of the subjects were collected. The actual intervention period was divided into two periods: Oct 28 - Nov 15, denoted as P2, and Nov 16 - Dec 5, denoted as P3. Performance in P2 and P3 was not statistically different, and so only their sum will be analyzed in the main text.

During these two intervention periods, the subjects were given feedback on their performance in the form of a monetary reward. The monetary reward was calculated as a function of the subject's activity and was divided according to the subject's experimental condition.

The game was not designed as a competition, and every subject had the potential to earn the maximal reward. That is, a previously non-active participant could gain the same reward as a highly active one, while the highly active person would need to work harder.

Measuring physical activities Physical activity measurement was based on accelerometer readings from the subjects' smartphones. Accelerometer scans were sampled in a duty cycle of 15 seconds every 2 min. During the 15 seconds, raw 3-axis accelerometer measurements were sampled at 5 Hz rate and combined to compute the vector magnitude for each sample. The variance of the magnitude in each one-second block was then computed [4]. The score was calculated by giving one point for every second, thresholded to three states (i) *still*, (ii) *moderate activity*, and (iii) *high activity*, where the two active levels were combined into a single active level. Participants were not constrained in the way they should carry the phone, but were told that the more they carry the phone on their body, the more of their activity would be accounted for their game score.

For analysis purposes, activity levels were normalized to the span of a single sample. For example, a normalized *daily average activity* is calculated by summing all accelerometer samples for the day and then dividing by the total count of accelerometer readings for the day. This gives the average activity level per reading for that day, and allows to easily do things like comparing between normalized average activity levels in different times of the day. It is trivial to convert a normalized value to actual time: for example, a normalized daily average value of 1 for an experimental group represents an average activity of 96 minutes per member.

Calculating rewards Game rewards were calculated every three days, using a reference window of the seven days preceding the current 3-day bin. Average and variance for daily activity count are calculated for the reference window, as well the daily average for the current 3-day bin. Reward depended solely on an individual's performance, and was mapped to be between \$0.50-\$5, in \$0.50 increments between one standard deviation above and below the reference average value. Values above or below the bounds received max or min value, respectively. To avoid discouragement due to zero reward, participants were not given less than 50 cents per reward period.

Cleaning the data As in [1], eleven subjects were removed from the study pool over the course of the intervention (due to prolonged technical issues that prevented reliable activity tracking, long durations of out of town travel, or dropping out of the longitudinal study entirely). For details on the final number of subjects in Peer See and Peer Reward condition, see Table S1.

As in [1], subject-day pairs that did not have sufficient information for generating a reliable average score for the day were precluded. For a single subject, a complete day's worth of data

Table S1: Numbers of participants per period/condition, and average number of days per user in parenthesis.

	Periods	Conditions	
		Group 2 (Peer-see)	Group 3 (Peer-reward)
P1	Oct 5 - Oct27 (23 days)	40 participants	41 participants
P2	Oct 28 - Nov 15 (42 days)		
P3	Nov 16- Dec 5 (62 days)		

consisted of 720 accelerometer score readings, since accelerometer scans were taken in two-minute intervals. Data was considered "missing" for an interval if there was no accelerometer score logged for that interval. When a person's day had fewer than 50% of the possible readings, that day was not used for the analysis and calculation of averages. Removed measurements accounted for less than 5.4% of the total available measurements.

3 The Reciprocity Survey

Human Subjects Approval The study was reviewed and approved by the Ethics Committee internal to the university where the study was performed. All participants provided a written consent (using an online form) to participate in this study and the Ethics Committee approved this consent procedure.

Data Description This data was collected as part of this study at a major Middle Eastern university during a four-months undergraduate course. 84 students in the class agreed to participate in the study (see details on demographics in Table S2).

Table S2: Demographics of participants in the reciprocity survey.

Gender	Male	Female								
	0.40	0.60								
Age	23	24	25	26	27	28	29	30	31	38
	0.01	0.19	0.25	0.21	0.18	0.08	0.02	0.01	0.02	0.01
Year of study	3rd	4th								
	0.96	0.04								

The study comprised a one-time computerized survey where each participant was asked

several demographic questions and three general questions about their perception of friendship. In addition, each participant was presented with a list of the 83 other participants (including their full name and their picture when available), and was asked the following four questions about his/her relationship with each one of the other participants:

1. How close are you to this person?

   0 - I do not know this person;
   1 - I recognize this person, but we never talked;
   2 - Acquaintance (we talk or hang out sometimes);
   3 - Friend;
   4 - Close friend;
   5 - One of my best friends.

2. Do you think that you have more friends than him/her? [Yes, No]
3. Do you think that most of your friends are also his/her friends? [Yes, No]
4. How close do you think this person considers you?

   0 - He/she does not know me;
   1 - He/she recognizes me, but we never talked;
   2 - Acquaintance (we talk or hang out sometimes);
   3 - Friend;
   4 - Close friend;
   5 - I am one of his/her best friends.

4 Directionality and Induced Peer Pressure

Our central hypotheses concern the relationship between the type of relationship subjects share with their buddies, on the susceptibility of increased physical activity under the experiment conditions. Several multivariate analyses are presented in tables S3, but the main story emerging from them is made clear in Fig. S2. In table S3 our dependent variable is the change in physical activity between the post-intervention phase (P2+P3) and the pre-intervention phase (P1) and the covariates consist of: (1) reciprocal friend - the number of buddies with whom the subject has a reciprocal friendship relationship (values 0 - 2); (2) alter-perceived friend - the number of buddies who were reported by the subject as friends but did not report the subject as friend (values 0 - 2); (3) ego-perceived friend - the number of buddies who reported the subject as friend but were not reported by the subject as friends (values 0 - 2); (4) tie strength - which is the sum of the nomination scores between the subject and the buddies; (5) Initial activity - which controls over the subject's pre-intervention activity levels. This can be described as follows: (6) gender (Male or Female); (7) same-gender - the number of buddies who had the same gender as the subject (values 0 - 2); (8) age; and (8) same-gender - the number of buddies who had the same ethnicity as the subject (values 0 - 2);

Table S3: Regression Coefficients for the change in activity under different experiment conditions

	Model 1	Model 2	Model 3	Model 4	Model 5	Model 6	Model 7	Model 8
Constant	0.05	0.03	-0.02	0.10	0.63^{***}	0.06	-0.01	0.56^{*}
	(0.08)	(0.08)	(0.09)	(0.09)	(0.11)	(0.29)	(0.09)	(0.27)
Reciprocal friend	0.44^{**}	0.45^{**}	0.45^{**}	0.44^{**}	0.30^{*}	0.44^{**}	0.45^{**}	0.33^{*}
	(0.16)	(0.16)	(0.16)	(0.16)	(0.13)	(0.16)	(0.16)	(0.14)
Alter perc. friend	0.15	0.15	0.18	0.16	0.12	0.15	0.16	0.13
	(0.13)	(0.14)	(0.14)	(0.14)	(0.11)	(0.14)	(0.13)	(0.12)
Ego perc. friend	0.31^{*}	0.30^{*}	0.31^{*}	0.29^{*}	0.24^{*}	0.31^{*}	0.32^{**}	0.24^{*}
	(0.12)	(0.12)	(0.12)	(0.12)	(0.10)	(0.12)	(0.12)	(0.10)
Tie Strength	$-0.04^{\cdot}$	$-0.04^{\cdot}$	$-0.04^{\cdot}$	$-0.04^{\cdot}$	$-0.03^{\cdot}$	$-0.04^{\cdot}$	-0.04^{*}	$-0.03^{\cdot}$
	(0.02)	(0.02)	(0.02)	(0.02)	(0.02)	(0.02)	(0.02)	(0.02)
Gender		0.05						0.08
		(0.08)						(0.07)
Same-gender			0.07					0.01
			(0.06)					(0.05)
Interv. group				-0.08				-0.01
				(0.08)				(0.07)
Pre-interv. activity					-0.43^{***}			-0.42^{***}
					(0.07)			(0.07)
Age						-0.00		0.00
						(0.01)		(0.01)
Same-ethnicity							0.08	0.03
							(0.05)	(0.05)
R^2	0.16	0.16	0.18	0.17	0.46	0.16	0.18	0.47
Adj. R^2	0.11	0.10	0.12	0.11	0.42	0.10	0.12	0.39
Num. obs.	76	76	76	76	76	76	76	76

Gender (Male = 1, Female = 0); dependent variables significance levels: $^{***}p < 0.001$, $^{**}p < 0.01$, $^{*}p < 0.05$, $^{\cdot}p < 0.1$

$$Y_i = \log\left(\frac{\text{Activity}(P2 + P3)}{\text{Activity}(P1)}\right) \sim N(\beta X_i, \sigma^2)$$

Where X_i represents the vector of demographic and experimental conditions of i with a constant variance σ^2.

The full specification model in Table S3 shows the results of the linear model analysis using a fairly wide specification of independent variables discussed above. We find that reciprocal ties are consistently associated with higher improvement in activity change and that this relationship persists even when we include detailed controls.

Both the gender of the subject and whether the buddies are from opposite gender had no significant association with the increase in activity. Controlling for a subject's age showed no effect on the change of activity as well. This was expected due to the highly homogeneous group

of participants (e.g., age $mean = 33$, $SD = 5$, both in years). A recent study by Aral et al., showed the importance of tie strength in moderating the effect of peer pressure [5]. In this work, we find the strength of the tie to be significant at 1% only, which highlights the importance of the tie 'type' rather than the tie 'strength'. Finally, the initial activity level coefficient is negative and statistically significant ($P < 0.001$). This suggests the difficulty of increasing the relative activity levels for already very active users in the post intervention period.

Previous studies have found that passive exposure to peers is sometimes sufficient to adopt a new health related behavior [e.g., [3, 6]]. This is particularly true when the behaviors to be transmitted are 'not difficult' and have low adoption barrier [e.g., registering for a health forum Web site [6] or signing up for an Internet-based diet diary [7]].

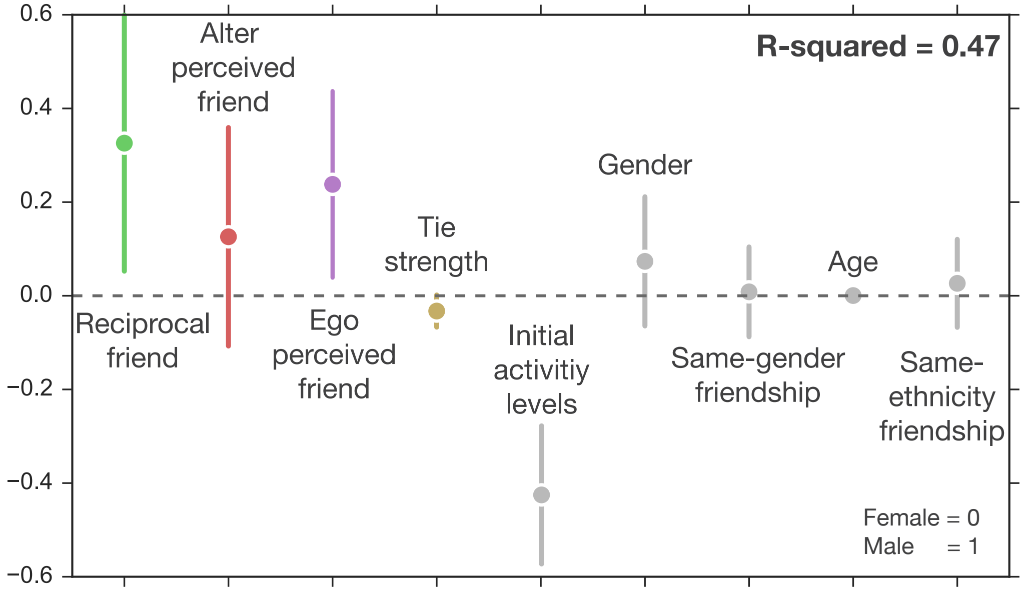

Figure S2: Change in physical activity under experiment conditions shows that the type of friendship is relevant to the effectiveness of the induced peer pressure while controlling over several covariates (gray lines and circles). The plot shows the mean effect size of the covariates (solid circles) and the 95% confidence intervals (bars).

We hypothesize that transmitting more complex behaviors (e.g., getting a vaccination, improving a diet, using condoms, or committing to physical exercises) might require people to engage in purposeful persuasion and therefore, the effects of interpersonal relationships will become more significant. Indeed, we see this as a trend in the current analysis, where the participants in the passive peer-see condition show no significant directionality effect while participants in the active peer-reward condition show a large directionality effect (see Table S4).

Table S4: Regression coefficients for the change in physical activity for peer-see and peer-reward intervention groups.

	Peer-See	Peer-Reward
Constant	0.02 (0.09)	0.11 (0.14)
Reciprocal friend	0.33 (0.20)	0.55 (0.27)*
Alter perceived friend	0.12 (0.16)	0.20 (0.23)
Ego perceived friend	0.24 (0.16)	0.36 (0.20)*
Tie Strength	−0.03 (0.02)	−0.05 (0.04)
R^2	0.13	0.16
Num. obs.	37	39

$^{***}p < 0.001$, $^{**}p < 0.05$, $^{*}p < 0.1$

5 Social Embeddedness

The Social Embeddedness (SE) of a pair of individuals captures the idea that the actions individuals choose are significantly refracted by the social relations within which they function [8]. We use six measures to examine the embeddedness within the joint neighborhood of nodes, namely the *Number of Common Friends*, the *Jaccard-Coefficent*, the *Adamic and Adar Score*, the *Preferential Attachment Score*, the *Edge Betweenness* and the *Clustering Coefficient*.

To that end, let $G_d = \langle V, E_d \rangle$ be the directed graph of the nominated friendships. That is, V is the set of nodes (i.e., experimental subjects) and E_d is the set of edges (i.e., friendship nominations). Stated differently, given two nodes $v_1, v_2 \in V$, an edge $(v_1, v_2) \in E_d$ means that the subject v_1 nominated subject v_2 as a friend. If E_d contains both of the edges (v_1, v_2) and (v_2, v_1), the friendship between v_1 and v_2 is *reciprocal*. Otherwise, the friendship between v_1 and v_2 is *unilateral*. The undirected graph $G_u = (V, E_u)$ represents the same topological structure of subjects and friendships, with the exception that the direction of edges is not specified. More formally, $E_u = \{\{v_1, v_2\} \mid (v_1, v_2) \in E_d \vee (v_2, v_1) \in E_d\}$ Finally, we define the undirected neighborhood of a node $v_1 \in V$, denoted by $\Gamma(v_1)$, as the set of nodes which are adjacent to v_1. Stated formally, $\Gamma(v_1) = \{v_2 \mid \{v_1, v_2\} \in E_u\}$.

We define the *number of common friends* between two nodes v_1 and v_2 as $|\Gamma(v_1) \cap \Gamma(v_2)|$. The logic for using the number of common friends to explain reciprocity is an extension to the network transitivity property. In other words, we hypothesize that given three nodes $v_1, v_2, v_3 \in V$, if E_u contains the three edges $\{v_1, v_2\}$, $\{v_1, v_3\}$ and $\{v_2, v_3\}$, then there is a higher probability that the three corresponding relationships are reciprocal. Fig. S3 shows the Empirical Cumulative Distribution Function (CDF) for the number of mutual friends between connected pairs. Indeed, we find that the probability that a node v_1 will form a reciprocal connection with node v_2 grows with the number of friends that v_1 and v_2 share in common.

The number of common friends can be a misleading indicator when one of the subjects is

highly connected. Therefore, we also compute the Jaccard-coefficent J, which normalizes the size of the common friends by the number of total friends. Conceptually, the Jaccard-coefficent captures the probability that a randomly selected node from the union of the friends of the two nodes, would result in a common friend of the two nodes. Stated formally, the Jaccard-coefficent is computed as follows:

$$J(v_1, v_2) = \frac{|\Gamma(v_1) \cap \Gamma(v_2)|}{|\Gamma(v_1) \cup \Gamma(v_2)|}$$

Note that although Liben-Nowell et al. [9] showed that the performance of the Jaccard-coefficient is worse than the number of common friends in the case of predicting the existence of edges; our findings for predicting the type of edges (i.e. reciprocal or unilateral) indicate that the Jaccard-coefficient provides a better signal. In Fig. **??**, we find that reciprocal friendships exhibit higher average in the Jaccard coefficient, which is expected as higher values could indicate higher embeddedness.

While the Jaccard-coefficent imposes some sort of a penalty to highly connected nodes, the Adamic and Adar score α gives more weight to low degree common friends (i.e., penalty to highly connected friends). This score was originally proposed as a metric for computing similarity between two web pages. For $|\Gamma(v_1) \cap \Gamma(v_2)| > 0$ the measure can be computed as follows:

$$\alpha(v_1, v_2) = \sum_{v_3 \in \Gamma(v_1) \cap \Gamma(v_2)} \frac{1}{log(|\Gamma(v_3)|)}$$

Fig. **??** shows the distribution of α for both, unilateral and reciprocal edges.

Another measure for embeddedness is the clustering coefficient C_t. It is defined as the number of triangles in which a node participates normalized by the maximum possible number of such triangles [10]. Although the original measure is used to quantify the clustering coefficient for a single node or the average value in the global graph, it is possible to compute a score for an edge between two nodes by multiplying the clustering coefficient of the two nodes [9]. Denoting the number of triangles around a node v as $t(v)$, the measure can be computed as follows:

$$C_t(v_1, v_2) = \frac{2t(v_1)}{|\Gamma(v_1)|\,(|\Gamma(v_1)| - 1)} \times \frac{2t(v_2)}{|\Gamma(v_2)|\,(|\Gamma(v_2)| - 1)}$$

Fig. S3 shows unilateral friendships exhibit lower average in clustering coefficient, which indicates a weaker intimacy when forming relationships.

We also compute the Preferential Attachment Score ω based on the well known *'rich gets richer'* model (see Fig. S3). The concept proposes that the probability of connecting two nodes is proportional to their degree. Newman [11] suggested using multiplication as an aggregation function for the two degrees. This suggestion is based on the work done by Newman [11] and Barabasi [12] on edge prediction for co-authorship networks. The measure is computed as follows:

$$\omega(v_1, v_2) = |\Gamma(v_1)| \times |\Gamma(v_2)|$$

Another useful measure — articulated in the context of sociology by Freeman [13] — is the *Edge Betweenness Centrality* e_b. This measure captures the total amount of flow across the edge assuming that the flow is evenly distributed on both nodes [14]. This can be computed as follows:

$$e_b(\{v_1, v_2\}) = \sum_{s,t \in V} \frac{\sigma(s, t|\{v_1, v_2\})}{\sigma(s, t)}$$

Where $\sigma(s, t)$ is number of all shortest paths and $\sigma(s, t|\{v_1, v_2\})$ is the number of those paths passing through the edge $\{v_1, v_2\}$.

We find that unilateral connections, generally, exhibit higher average, median, and maximum Edge Betweenness score (see Fig. S3). This is not surprising, as edges connecting nodes from different communities could be an indicator for weaker relationship.

6 Social Centrality

It is known that hierarchical social organization is highly characterized by acts of subordination and dominance. In particular, group organization is shaped by ordered, linearly transitive social relationships and adaptive advantages of divisions of labor and social roles [15].

In this work we used four common measures to capture the Social Centrality (SC) of a node in the network, namely, *Degree Centrality*, *Closeness Centrality*, *Eigenvector Centrality Betweenness Centrality*.

Given a relationship between two nodes, we compute each one of the centrality measures for each dyad and take the difference, denoted by ΔC_d, ΔC_c, ΔC_b, and ΔC_e.

The degree centrality C_d for a node v_1 is the fraction of nodes it is connected to. This can be formulated as follows:

$$C_d(v_1) = \frac{|\Gamma(v_1)|}{|V|}$$

The closeness centrality C_c for a node v_1 measures the inverse of the sum of the shortest path distances between v and all other nodes normalized by the sum of minimum possible distances. This can be formulated as follows:

$$C_c(v_1) = \frac{n - 1}{\sum\limits_{v_2 \in V} \sigma(v_1, v_2)}$$

where $\sigma(v_1, v_2)$ is the length of the shortest path between v_1 and v_2, and n is the number of nodes in the connected part of the graph containing the node v_1.

The betweenness centrality C_b for a node v_1 is similar to the edge betweenness, however, computed for each node independently.

$$C_b(v_1) = \sum_{s,t \in V} \frac{\sigma(s, t|v_1)}{\sigma(s, t)}$$

Where $\sigma(s,t)$ is the number of shortest paths and $\sigma(s,t|v_1)$ is the number of those paths passing through node v_1.

The eigenvector centrality C_e uses the power method to find the eigenvector for the largest eigenvalue of the adjacency matrix of G_u (see [16]).

Fig. S4 shows the average difference in Social Centrality (SC) between pairs. We find that larger differences seem to indicate lower likelihood for reciprocity.

While SC features take a purely dyadic view of friendship tie formulation, according to which the friendship strength is determined only by the characteristics of the individuals it connects, SE features take into account the global view in which tie strength is driven by the entire network topology.

7 Predicting Reciprocity and Directionality

We construct models and analyze the predictive power of the identified Social Embeddedness and Social Centrality measures in two classification tasks: first, predicting reciprocity (i.e., reciprocal vs unilateral ties), and second, predicting directionality (i.e., direction of unilateral ties).

For each classification task, we train and test a classifier in K-fold cross-validation ($K = 10$), to ensure that test observations were not used during training. In both cases, we report the summarized sensitivity and specificity of the classifier using the average area under the receiver-operator characteristic curve (AUC). We also calculate a 95% confidence interval (CI) by Bootstrap re-sampling for comparison (calculated using 10,000 bootstraps). We compare the results to the *baseline* classifier, which simply predicts the majority class as a benchmark for the trained classifier (and which always produces 0.5 AUC score).

First, we used a simple Logistic Regression classifier and a single feature for each classification task (i.e. number of common friends for the first task and difference in degree centrality for the second task). When trying to identify reciprocal ties, the Logistic Regression classifier performed significantly better than the baseline obtaining 0.81 AUC (95% CI: 0.77 – 0.85). As for identifying the direction of unilateral ties, the Logistic Regression classifier performed better than the baseline, although the results obtained were less compelling: an average AUC of 0.61 (95% CI: 0.58 – 0.66).

Finally, we used all 10 investigated features (6 SE features and 4 SC features as described in the *Supplementary Materials*) to train a Random Forest classifier for each classification task, obtaining encouraging results for both classification tasks: an average AUC of 0.85 (95% CI: 0.82–0.87) for the reciprocal classification task and an average AUC of 0.75 (95% CI: 0.70–0.80) for the directionality task. The performances of the two classification tasks are reported in Fig. S5.

Single Features Performance For each identified Social Embeddedness feature, we use a simple Logistic Regression classifier to predict the reciprocity of a tie. Training and testing

are done in 10-fold cross-validation and we report the average AUC for each of the features in Fig. S6.

Similarly, for each identified Social Centrality feature, we use a simple Logistic Regression classifier to predict the directionality of a unilateral tie. Training and testing are done in 10-fold cross-validation and we report the average AUC for each of the features in Fig. S7.

8 Reciprocal Ties and Incoming Edges Factors

In this section, we explore the effect of Social Embededdness (SE) and Social Centrality (SC) on the Probability of an ego to form a reciprocal tie or be perceived as a friend (i.e., incoming edge) in the Friends and Family dataset. Control variables include whether the dyad have the same country of origin, ethnicity, and gender. In both cases, we use a logit model for the friendship ties in the network as follows:

$$Y_i = Bernoulli(\pi_i)$$
$$\pi_i = \frac{1}{1 + e^{-X_i \beta}}$$

Where X_i represents the vector of control variables including whether the dyad have the same country of origin (values 0 or 1), ethnicity (values 0 or 1), and gender (values 0 or 1).

Where for the SE (Recall that $|\Gamma(v_1) \cap \Gamma(v_2)|$ is the number of common friends):

$$X_i\beta = \beta_0 + X_{i,gender}\beta_1 + X_{i,ethnicity}\beta_2 + X_{i,origin}\beta_3 + X_{i,|\Gamma(v_1)\cap\Gamma(v_2)|}\beta_4$$

While for the SC logit model, we incorporate the difference in degree centrality ΔC_d:

$$X_i\beta = \beta_0 + X_{i,gender}\beta_1 + X_{i,ethnicity}\beta_2 + X_{i,origin}\beta_3 + X_{i,\Delta C_d}\beta_4$$

Table S5 shows the coefficients for both models. In order to compute the expected probabilities of forming reciprocal ties or incoming-ties for each number of common friends or difference in centrality value, we simulate $\tilde{\beta}$ from the sampling distribution of the estimated $\hat{\beta}$:

$$\tilde{\beta} \sim MVN(\hat{\beta}, \hat{V}(\hat{\beta}))$$

Where $\hat{V}(\hat{\beta})$ is the variance covariance matrix. We then choose one value for the explanatory variable (i.e., specific number of common friends) and hold all other covariates at their median value and donate the vector of values X_c. Using X_c and $\tilde{\beta}$, we calculate the systematic component $\tilde{\pi}$ as:

$$\tilde{\pi} = \frac{1}{1 + e^{-X_c\tilde{\beta}}}$$

Then for each $\tilde{\pi}$ draw, we simulate from the stochastic component:

$$\tilde{Y_c} = Bernoulli(\tilde{\pi})$$

Then we report the mean of these simulations for each $\tilde{\pi}$, $E[Y_C]$ and the 95% confidence interval using the 2.5% and 97.5% quantiles from 10,000 simulations.

Table S5: Logit coefficients for the reciprocal ties and incoming unilateral tie.

	Reciprocal ties	Incoming ties
Number of common friends	0.19^{***}	
	(0.02)	
Same gender	0.10	-0.03
	(0.19)	(0.21)
Same country of origin	1.08^{***}	0.37
	(0.26)	(0.26)
Same religion	0.39	0.32
	(0.23)	(0.25)
Same ethnic group	-0.09	-0.27
	(0.23)	(0.25)
$\Delta degree$		-3.89^{***}
		(1.08)
(Intercept)	-2.61^{***}	-0.28
	(0.23)	(0.19)
BIC	737.81	548.05
Log Likelihood	-349.26	-256.18
Deviance	698.52	512.36
Num. obs.	698	383

$^{***}p < 0.001$, $^{**}p < 0.01$, $^{*}p < 0.05$

9 Additional Datasets

We repeat some of the analyses we performed on the Friends and Family dataset using additional datasets with the aim to verify whether our findings have a more general validity. We consider datasets collected in the US, Europe, and the Middle East to take into account the possible effects of cultural differences.

Reality Mining This dataset was collected in 2004 in the US [2]. The goal of this study was to explore the capabilities of the smart phones that enabled social scientists to investigate human interactions beyond the traditional survey-based or simulation-based methodology. The subjects were 75 students or faculty in the MIT Media Lab, and 25 incoming students at the MIT Sloan business school adjacent to the Media Lab. Out of the 75 Media Lab participants, 20 were incoming masters students and 5 were incoming MIT freshman, and the rest had remained in the Media Lab for at least a year. In a survey at the end of the collection period, participants have been asked who they usually spend time with, both in the workplace and out of the workplace, and who they would consider to be within their circle of friends (providing just a binary indication of friends/not friends).

Strongest Ties This study took place during a full semester course (13 weeks) at a large European university and was designed to measure human interactions across a variety of communication channels [17]. Specifically, data was collected on face-to-face interactions, telecommunication, social networks, location, and background information (personality, demographic, health, politics) for a densely connected population, using state-of-art smartphones as social sensors. Participants were students in an advanced course, involving work with high-level programming, data modelling, and simple machine learning. At the beginning of the course, 80 out of the 95 students agreed to participate in the study. Participants were asked to rate how well they knew all the other participants, using a 12-points grading scale: 0 - I do not know this person; 2 - I recognize this person, but we never talked; 4 - Acquaintance (we talk or hang out sometimes); 7 - Friend; 10 - Close friend; 12 - One of my best friends.

Social Evolution This dataset was collected in 2008 in the US [18]. The study was designed to investigate the adoption of political opinions, diet, exercise, obesity, eating habits, epidemiological contagion, depression and stress, dorm political issues, interpersonal relationships, and privacy. The data collection covered the locations, proximities, and phone calls of more than 80% of residents who lived in the dormitory used in the Social Evolution experiment, as captured by their cell phones from October 2008 to May 2009. This dormitory had a population of approximately 30 freshmen, 20 sophomores, 10 juniors, 10 seniors and 10 graduate student tutors. Friendship surveys were collected monthly, asking participants to indicate whether they share a relationship with another participant at the time of survey. The surveyed relationships (that we consider as friendships) were: “close friends”, “participated in at least two common activities per week”, “discussed politics since the last survey”, “shared all tagged Facebook photos”, “shared blog/live journal/Twitter activities”.

Personality Survey This data was collected as part of a research project in a large Middle Eastern university during an undergraduate course at the Information Systems Engineering department. 90 students in the class agreed to participate in the survey. The survey comprised questions about personality, empathy, technical proficiency, and friendship. Part of the survey was a self-reported questionnaire on students’ friendship relationship with the other students in the class. Each participant was asked to score other participants on a 0–5 scale: 0 - I do not know this person; 1 - I recognize this person, but we never talked; 2 - Acquaintance (we talk or hang out sometimes); 3 - Friend; 4 - Close friend; 5 - One of my best friends.

Not All Friendships Are Reciprocal The participants of all the considered studies were asked to indicate their friendship relationships and the closeness with other participants through self-reported surveys. Fig. S8 shows that for the additional datasets, as well as in the Friends and Family study, the percentage of reciprocal ties is below 55%. This supports our hypothesis that generally not all friendships are reciprocal.

Predicting Reciprocity and Directionality In this study, we investigated the predictability of reciprocity and directionality of ties in the Friends and Family dataset based on the topological structure of the undirected and unweighted social network. We were interested in two classification tasks: (1) predicting the type of a friendship relationship, i.e. reciprocal or unilateral, (2) predicting the directionality of a friendship tie, i.e. incoming or outgoing. We used 10 features belonging to two categories—Social Embeddedness and Social Centrality—to train a Random Forest classifier for each classification task, and tested it on the Friends and Family dataset obtaining encouraging results: an average AUC of 0.85 (95% CI: 0.82–0.87) for the first classification task and an average AUC of 0.75 (95% CI: 0.70–0.80) for the second.

We repeat the evaluation of both classification tasks on additional datasets (table S6). We obtain results very close to the ones with the Friends and Family dataset for all additional datasets, with a noticeable drop in performance only for the Reality Mining and Reciprocity Survey datasets. In the first classification task (reciprocal vs unilateral, Fig. S9) the average AUC for the additional datasets (0.78 on average) is generally slightly lower than in the Friends and Family dataset (0.85). On the contrary, in the second classification task (incoming vs outgoing, Fig. S9) the average AUC for the additional datasets (0.89 on average) is generally higher than in the Friends and Family (0.75).

Table S6: Evaluation results of reciprocity and directionality classification on additional datasets.

Dataset	Reciprocal vs Unilateral		Incoming vs Outgoing	
	Average AUC	95% C.I.	Average AUC	95% C.I.
Friends and Family	0.85	[0.82–0.87]	0.75	[0.70–0.80]
Strongest Ties	0.78	[0.72–0.83]	0.80	[0.72–0.88]
Reality Mining	0.57	[0.46–0.64]	0.71	[0.47–0.86]
Social Evolution 2008-09-09	0.77	[0.70–0.78]	0.97	[0.96–0.98]
Social Evolution 2008-10-19	0.81	[0.78–0.84]	0.96	[0.94–0.97]
Social Evolution 2008-12-13	0.78	[0.76–0.80]	0.94	[0.93–0.95]
Social Evolution 2009-03-05	0.83	[0.80–0.84]	0.96	[0.96–0.97]
Social Evolution 2009-04-17	0.83	[0.79–0.85]	0.97	[0.96–0.98]
Social Evolution 2009-05-18	0.81	[0.79–0.83]	0.97	[0.96–0.98]
Personality Survey	0.90	[0.89–0.91]	0.96	[0.95–0.97]
Reciprocity Survey	0.73	[0.69–0.76]	0.69	[0.63–0.76]

References and Notes

[1] Nadav Aharony, Wei Pan, Cory Ip, Inas Khayal, and Alex Pentland. Social fmri: Investigating and shaping social mechanisms in the real world. *Pervasive and Mobile Computing*,

7(6):643–659, 2011.

[2] Nathan Eagle and Alex (Sandy) Pentland. Reality mining: sensing complex social systems. *Personal Ubiquitous Comput.*, 10(4):255–268, 2006.

[3] Anmol Madan, Sai T Moturu, David Lazer, and Alex Sandy Pentland. Social sensing: obesity, unhealthy eating and exercise in face-to-face networks. In *Wireless Health 2010*, pages 104–110. ACM, 2010.

[4] Roger G Eston, Ann V Rowlands, and David K Ingledew. Validity of heart rate, pedometry, and accelerometry for predicting the energy cost of children's activities. *Journal of applied physiology*, 84(1):362–371, 1998.

[5] Sinan Aral and Dylan Walker. Tie strength, embeddedness and social influence: A large scale networked experiment. *Management Science*, 2014.

[6] Damon Centola. The spread of behavior in an online social network experiment. *science*, 329(5996):1194–1197, 2010.

[7] Damon Centola. An experimental study of homophily in the adoption of health behavior. *Science*, 334(6060):1269–1272, 2011.

[8] Mark Granovetter. Economic action and social structure: The problem of embeddedness. *American Journal of Sociology*, pages 481–510, 1985.

[9] David Liben-Nowell and Jon Kleinberg. The link-prediction problem for social networks. *J. Am. Soc. Inf. Sci. Technol.*, 58(7):1019–1031, May 2007.

[10] J. Saramäki, M. Kivelä, J.P. Onnela, K. Kaski, and J. Kertesz. Generalizations of the clustering coefficient to weighted complex networks. *Physical Review E*, 75(2):027105, 2007.

[11] M.E.J. Newman. Clustering and preferential attachment in growing networks. *Physical Review E*, 64(2):025102, 2001.

[12] A. L. Barabási, H. Jeong, Z. Néda, E. Ravasz, A. Schubert, and T. Vicsek. Evolution of the social network of scientific collaborations. *Physica A: Statistical Mechanics and its Applications*, 311(3-4):590 – 614, 2002.

[13] Linton C. Freeman. A Set of Measures of Centrality Based on Betweenness. *Sociometry*, 40(1):35–41, March 1977.

[14] C.C. Aggarwal. *Social Network Data Analytics*. Springer, 2011.

[15] Kingsley Davis and Wilbert E Moore. Some principles of stratification. *American Sociological Review*, 10:242, 1945.

[16] Ulrik Brandes and Thomas Erlebach. *Network Analysis: Methodological Foundations (Lecture Notes in Computer Science)*. Springer-Verlag New York, Inc., Secaucus, NJ, USA, 2005.

[17] Yves-Alexandre de Montjoye, Arkadiusz Stopczynski, Erez Shmueli, Alex Pentland, and Sune Lehmann. The strength of the strongest ties in collaborative problem solving. *Scientific reports*, 4, 2014.

[18] Anmol Madan, Manuel Cebrian, Sai Moturu, Katayoun Farrahi, and Alex Pentland. Sensing the" health state" of a community. *IEEE Pervasive Computing*, 11(4):36–45, 2012.

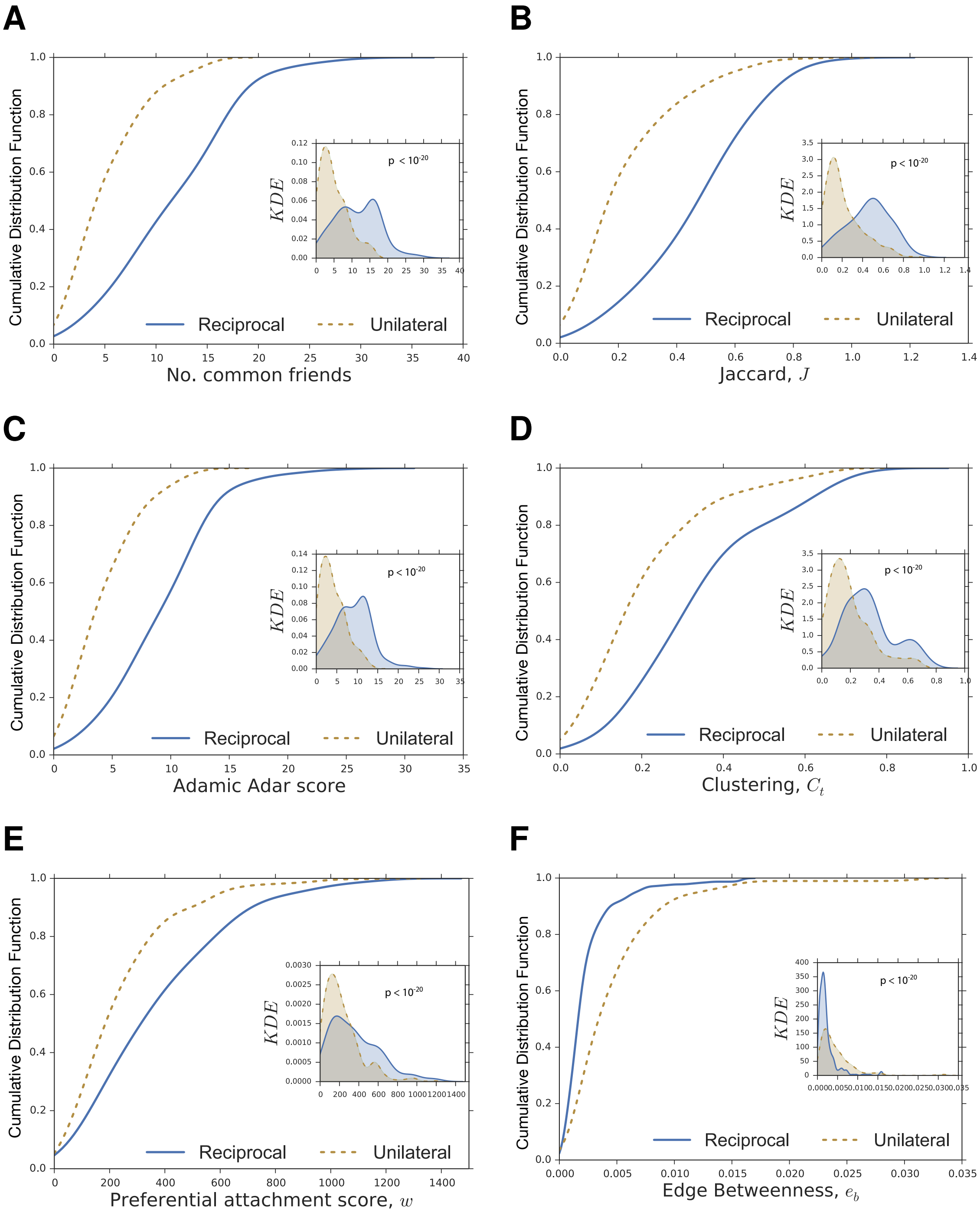

Figure S3: Social Embeddedness features show high discrepancies between reciprocal and unilateral edges. These discrepancies can be used to distinguish the type of tie from undirected networks.

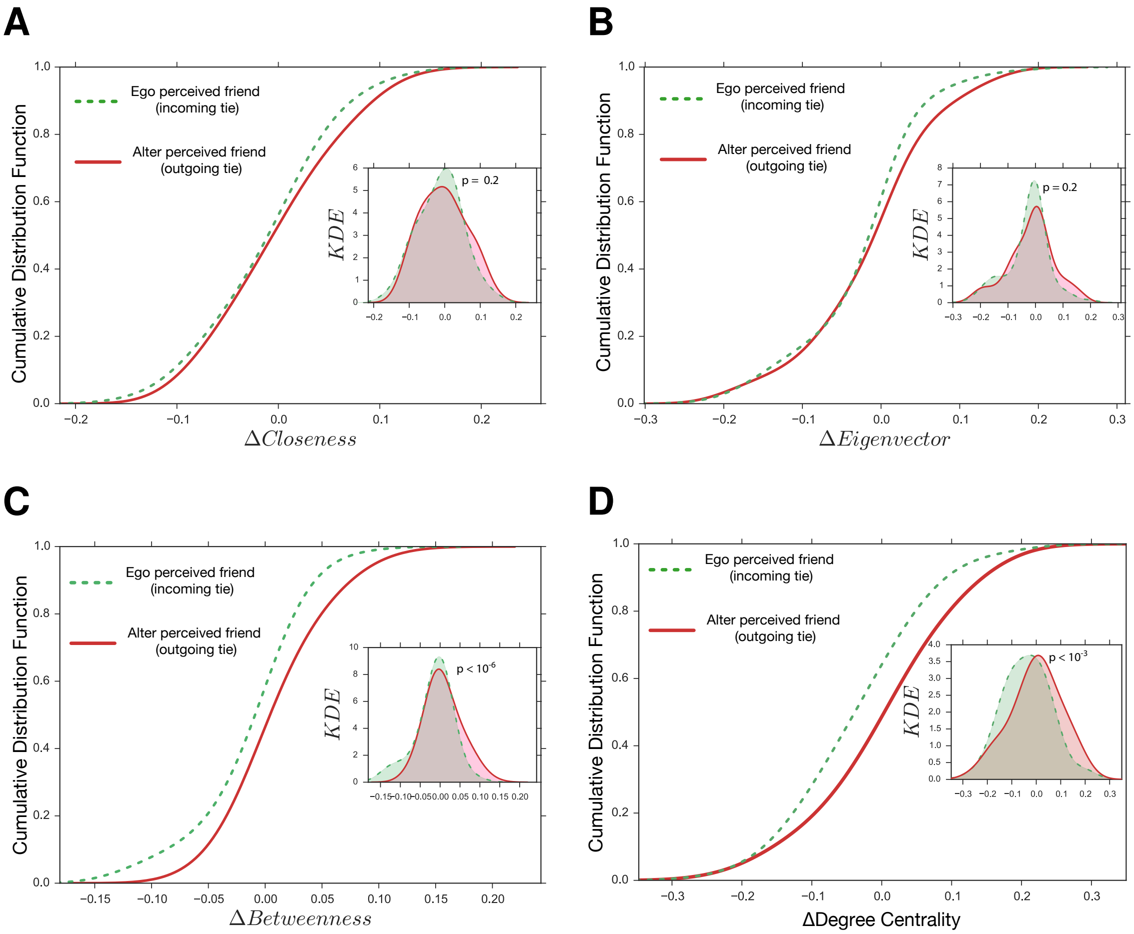

Figure S4: Social Centrality features show discrepancies between reciprocal and unilateral edges.

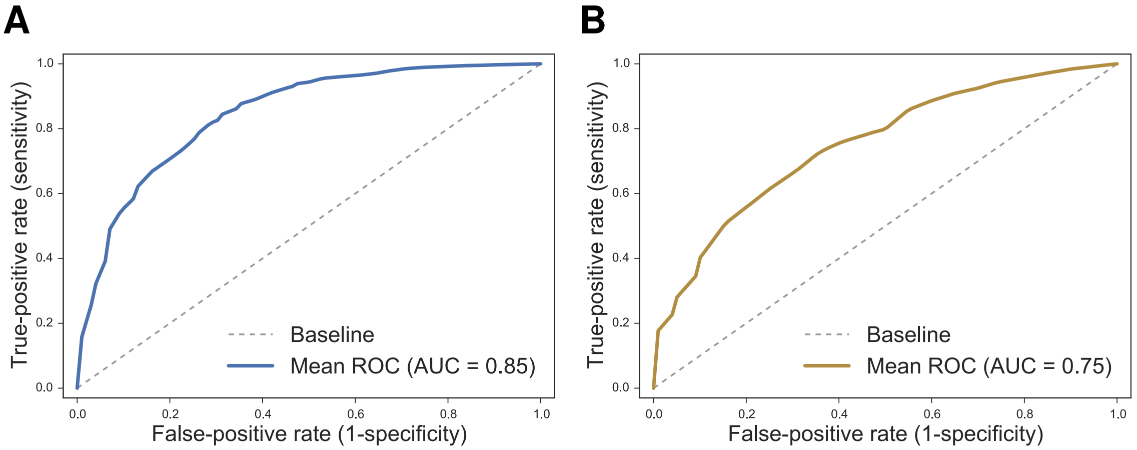

Figure S5: Mean ROC curves demonstrating the model performance in predicting ties type. Panel (**A**) shows the prediction performance for reciprocal ties (AUC = 0.85, 95% CI: 0.82–0.87). Panel (**B**) shows the model performance in prediction incoming ties (AUC = 0.75, 95% CI: 0.70–0.80).

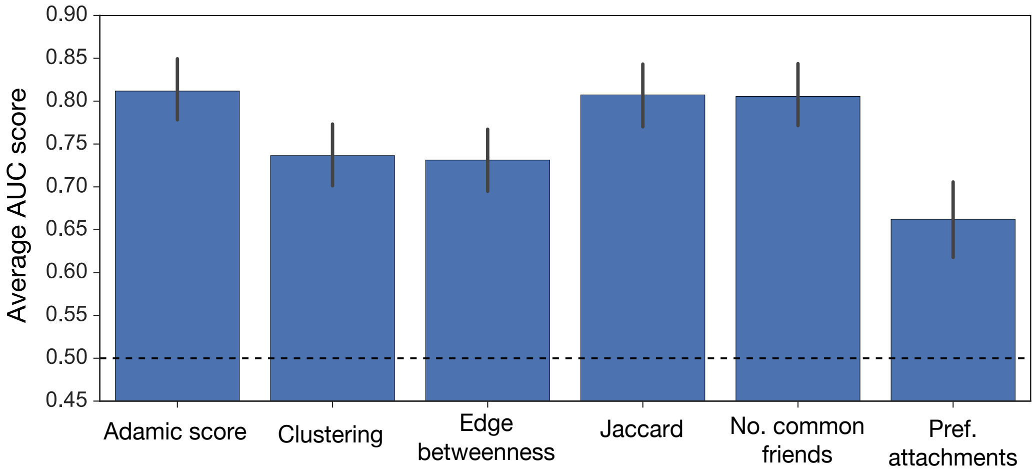

Figure S6: The y-axis represents the area under the receiver-operating characteristic curve and the error bars represent the 95% confidence intervals. Confidence intervals are constructed using the 2.5% and 97.5% quantiles from 1000 bootstrap replicates. The dashed horizontal line is the baseline.

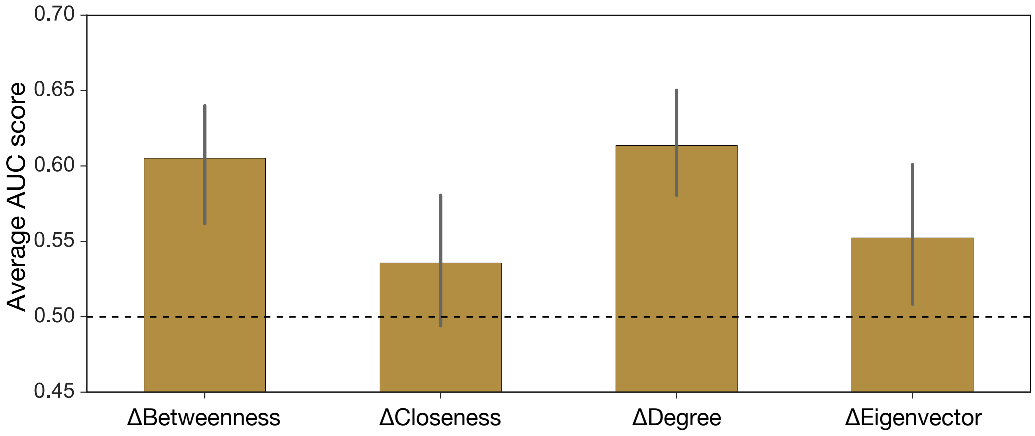

Figure S7: The y-axis represents the area under the receiver-operating characteristic curve and the error bars represent the 95% confidence intervals (calculated via 10^3 bootstraps). The dashed horizontal line is the baseline.

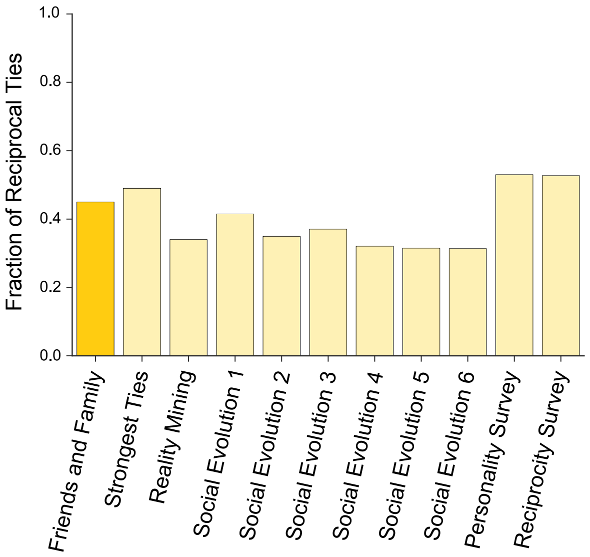

Figure S8: Fraction of reciprocal ties in additional datasets. The fraction of reciprocal ties for the Friends and Family study is reported (dark blue) next to the fraction observed in additional datasets (light blue): *Reality Mining*, *Strongest Ties*, *Social Evolution* study, a *Personality Survey*, and our *Reciprocity Survey*. The *Social Evolution* study is split into six temporal slices, and we report each of them as a separate dataset. The average percentage of reciprocal tie for the entire *Social Evolution* study is 35%.

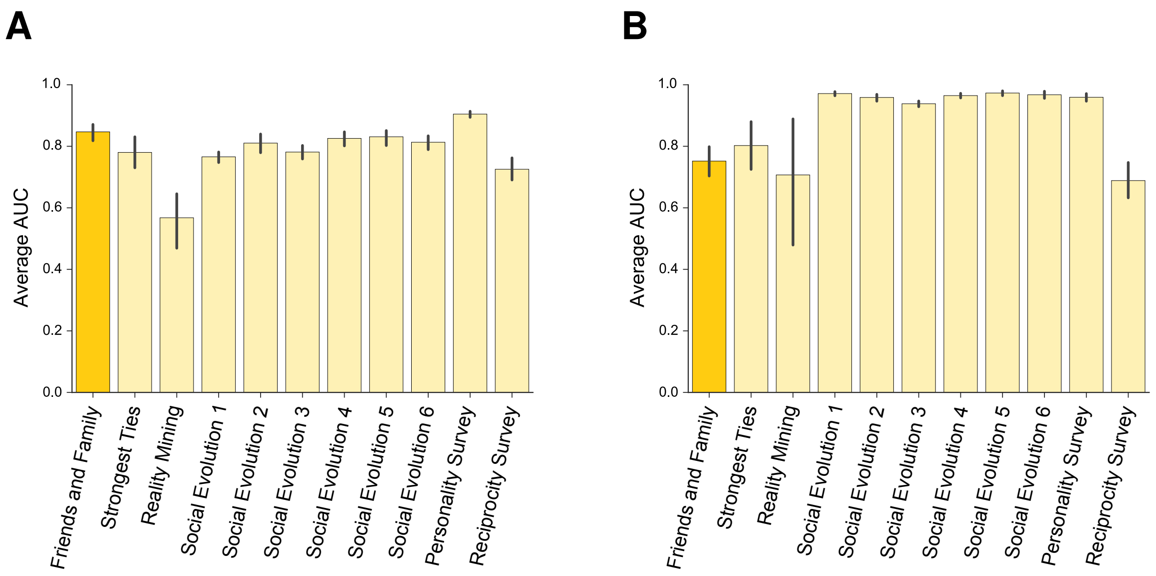

Figure S9: Comparison of classification results with additional datasets. We repeat the two classification tasks (reciprocal vs unilateral (**A**), incoming vs outgoing (**B**)) on additional datasets and report the results for the Friends and Family study (dark yellow) next to the additional datasets (light yellow): *Reality Mining*, *Strongest Ties*, *Social Evolution* study, a *Personality Survey*, and our *Reciprocity Survey*. The *Social Evolution* study is split into six temporal slices, and we report each of them as a separate dataset. We report the average AUC of the results provided by a Random Forest evaluated with a 10-fold cross-validation method and the confidence intervals at 95% computed via bootstrapping.
